# miRDM-rfGA: Genetic algorithm-based identification of a miRNA set for detecting type 2 diabetes

**DOI:** 10.1186/s12920-023-01636-2

**Published:** 2023-08-22

**Authors:** Aron Park, Seungyoon Nam

**Affiliations:** 1 Department of Health Sciences and Technology, Gachon Advanced Institute for Health Sciences and Technology (GAIHST), Gachon University, Incheon, 21999 Korea; 2 Department of Genome Medicine and Science, AI Convergence Center for Medical Science, Gachon University Gil Medical Center, Gachon University College of Medicine, Incheon, 21565 Korea

**Keywords:** Genetic algorithm, Feature selection, miRNA, Type 2 diabetes, Biomarker discovery

## Abstract

**Background:**

Type 2 diabetes mellitus (T2DM) affects approximately 451 million adults globally. In this study, we identified the optimal combination of marker candidates for detecting T2DM using miRNA-Seq data from 95 samples including T2DM and healthy individuals.

**Methods:**

We utilized the genetic algorithm (GA) in the discovery of an optimal miRNA biomarker set. We discovered miRNA subsets consisting of three miRNAs for detecting T2DM by random forest-based GA (miRDM-rfGA) as a feature selection algorithm and created six GA parameter settings and three settings using traditional feature selection methods (F-test and Lasso). We then evaluated the prediction performance to detect T2DM in the miRNA subsets derived from each setting.

**Results:**

The miRNA subset in setting 5 using miRDM-rfGA performed the best in detecting T2DM (mean AUROC = 0.92). Target mRNA identification and functional enrichment analysis of the best miRNA subset (hsa-miR-125b-5p, hsa-miR-7-5p, and hsa-let-7b-5p) validated that this combination was involved in T2DM. We also confirmed that the targeted genes were negatively correlated with the clinical variables related to T2DM in the BxD mouse genetic reference population database.

**Conclusions:**

Using GA in miRNA-Seq data, we identified the optimal miRNA biomarker set for T2DM detection. GA can be a useful tool for biomarker discovery and drug-target identification.

**Supplementary Information:**

The online version contains supplementary material available at 10.1186/s12920-023-01636-2.

## Background

Type 2 diabetes mellitus (T2DM) is characterized by abnormalities in carbohydrate, lipid, and protein metabolism pathways. Dysregulation of insulin secretion and response in T2DM results in hyperglycemia.

According to the International Diabetes Federation, 451 million adults worldwide have diabetes; this number is expected to reach 693 million by 2045 [1]. Diabetes is among the top 10 causes of death globally, and the risk of all-cause mortality increases by approximately 2–threefold among individuals with diabetes [2].

T2DM is a representative disease that leads to diabetic nephropathy, retinopathy, neuropathy, and other complications, including colon and liver cancers [3–9].

The main objective of this study was to identify the optimal biomarkers for detecting T2DM and identifying drug targets to treat T2DM. Among the various putative drug targets, epigenetic mechanisms such as microRNAs (miRNAs) may contribute to the development of common diseases, including T2DM [10, 11]. miRNAs, which are short (~ 22 nucleotides) noncoding RNAs, have emerged as key cell type-specific regulators of gene expression, operating primarily to inhibit target genes post-transcription by binding with complementary mRNA [12].

In T2DM, miRNAs target various genes related to glucose and fatty acid metabolism and the insulin signaling pathway in diverse tissues (e.g., skeletal muscle, pancreas, adipocytes, and liver), thereby affecting physiological functions [10, 13, 14].

Because of the importance of the regulation of miRNAs in T2DM, several studies have tried to identify miRNA biomarkers for T2DM by characterizing differentially expressed miRNAs (in blood, pancreas, adipocytes, skeletal muscle, and liver) in T2DM patients [3–9, 15–19]. In addition, miRNA biomarker discovery has confirmed the negative correlation between the expression of discovered miRNAs and their target mRNAs [3–11, 14–19].

Biomarker discovery based on feature selection and applying the machine learning method is a promising approach [20–25]. However, these methods use genetic data to create a model that classifies T2DM patients and to derive important features that affect the classification performance of the model. In this case, it is possible to calculate the importance of miRNAs that affect disease diagnosis. However, the optimal number of entries in the biomarker combination for disease diagnosis is difficult to define using these methods. In other words, it is difficult to determine an optimal combination of marker candidates. As marker candidates belong to differentially expressed (DE) genes (or miRNAs), and DE genes are usually numerous, identification of the best combination of select markers is challenging [26]. Even though the combination of machine learning and genetic algorithm (GA) techniques can be adopted for addressing these challenges, it still has not been widely explored in the context of T2DM biomarker discovery.

To identify an optimal miRNA set for T2DM, we integrated GA and random forest to develop a novel feature selection algorithm. We first obtained public miRNA-Seq datasets from T2DM patients and healthy controls (HC) from the Gene Expression Omnibus (GEO). We then compared diverse feature selection methods (i.e., F-test in analysis of variance [ANOVA] and least absolute shrinkage and selection operator [Lasso]) under three settings with our GA-based feature selection (named miRDM-rfGA) under six settings. In each setting, we evaluated the performance of each biomarker set in detecting T2DM. We then obtained publicly available mouse phenotype data to show biological associations between T2DM-related phenotypes and the biomarker set [27]. In this study, we demonstrated the utility of GA for the discovery of an optimal miRNA biomarker set. This study not only emphasizes the significance of miRNAs in T2DM but also provides the novel application of GA and machine learning techniques in the discovery of optimal combinations of disease biomarkers.

## Materials and methods

### Data collection

**Fig. 1:**
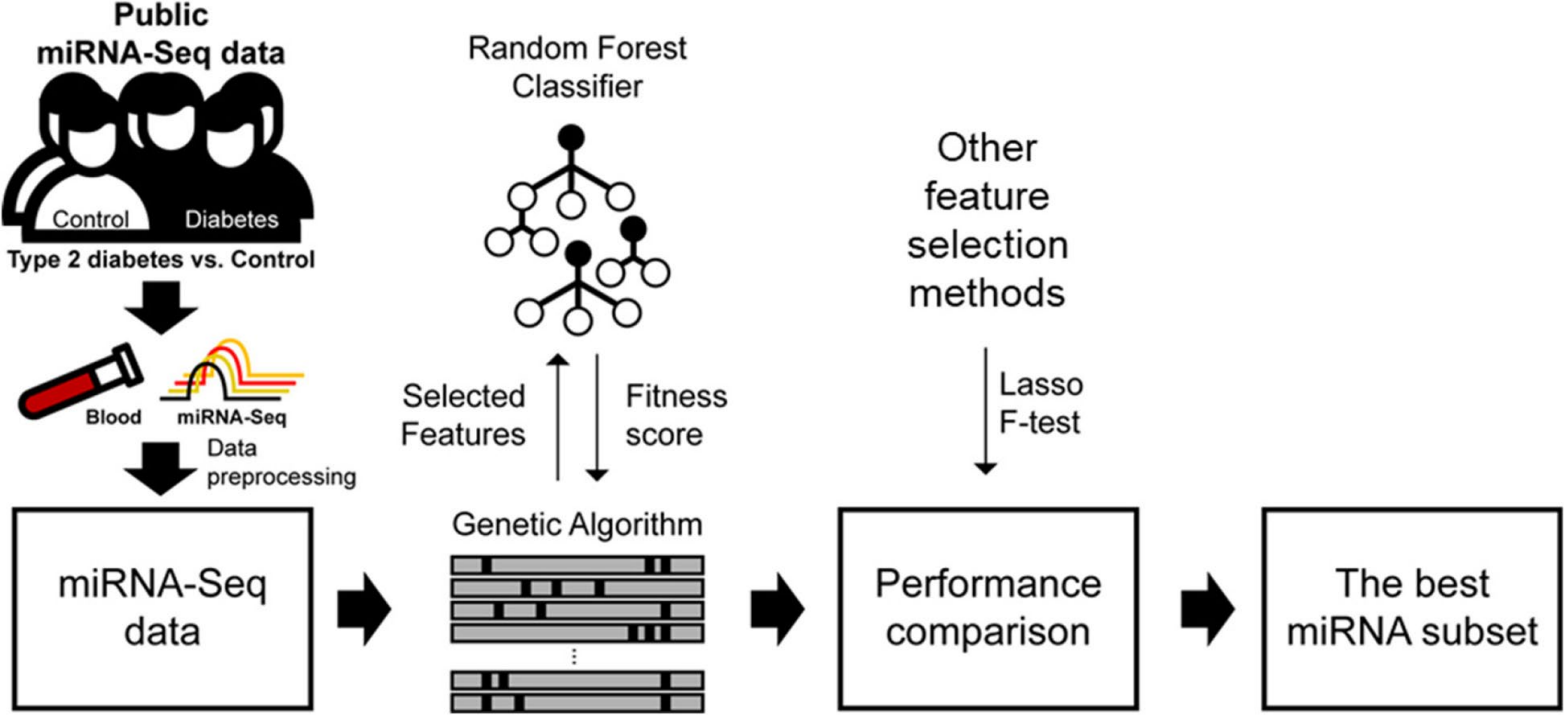
Overview of this study. T2DM and HC samples in blood were collected from NCBI GEO. Feature selection using GA with RF was used for miRNA biomarker discovery. We also compared the classification performance of T2DM with other traditional feature selection methods (F-test in ANOVA and Lasso)

We searched for and acquired public miRNA-Seq data in the blood tissue for biomarker discovery (Fig. 1). First, we accessed Sequence Read Archive (SRA) [28] and obtained relevant datasets by using the search terms “Diabetes” and “miRNA”. After acquiring the results from the query, two datasets (SRP151126 and SRP093728) of miRNA profiling related to T2DM in human blood were obtained (Supplementary Figure S1 and Supplementary Table S1). Thus, we selected 95 samples (56 HCs and 39 T2DM patients) to download fastq sequences and perform further analyses (Supplementary Table S1).

### Pre-processing of miRNA-Seq data and dataset preparation

For the 95 miRNA-Seq samples, we pre-processed the data using FastQC v0.11.7 (http://www.bioinformatics.babraham.ac.uk/projects/fastqc/) and Cutadapt v1.3 [29]. Samples with a Phred quality score of less than 20 were removed. We used Illumina Universal Adapter and Illumina Small RNA 3’ Adapter for adapter trimming. Subsequently, we trimmed the reads with lengths of 18–25 bp. The miRdeep2 mapper (v 2.0.0.8) [30] was used for sequence alignment of the human reference genome version hg19, and miRNAs were annotated using miRbase version 21 [31]. For quantifying the miRNA expression, reads per million (RPM) were used and Z-normalization was used to display the miRNA expression.

To remove sparse miRNAs, miRNAs with a ratio of 90% or more in a sample with an expression value of 0 for each miRNA from a total of 95 samples were removed, yielding a total of 1169 miRNAs.

The training and test sets were created in a ratio of 7:3. The training set was used for biomarker discovery using the GA or traditional feature selection method, and the test set was used to evaluate the performance of the T2DM patient classification model using biomarkers selected by each method.

### Biomarker set discovery using GA and traditional feature selection methods

According to previous studies [32–36], the optimal number of entries in biomarker sets range from two to nine. Inspired by a previous study using three miRNAs as biomarker combinations [36], we aimed to discover an optimal biomarker set consisting of three miRNAs for detecting T2DM. Therefore, we used the feature selection method to identify a biomarker set comprising three miRNAs. To this end, GA was adopted, and we used the F-test in ANOVA and Lasso as a traditional feature selection method for comparison with GA.

GA is a heuristic algorithm that uses natural selection to find the best solution [37–39]. To simulate natural selection, the GA generates a population and a set of individuals (i.e., chromosomes and solutions). Individuals consist of a set of features (or genes) that represent a set of solutions to a predefined problem.

For each individual, the fitness score was calculated to show how each solution, indicating selected features, was optimal. The best individual (i.e., the best solution) in the population was selected and included in a new population for the next generation, and the other individuals were produced by crossover and mutation. Thus, the GA generates individuals repeatedly, assesses their fitness, and terminates when the given goal is met or when some stopping criteria are met.

In this study, we implemented a random forest (RF)-based GA feature selection algorithm (miRDM-rfGA) with miRNA-Seq data as input, and determined the three most optimal miRNA subsets evaluated by a fitness score using RF.

For miRDM-rfGA, five phases were considered: (1) generation of the initial population, (2) evaluation of each individual by a fitness function that explains whether the solution is good or not, (3) selection of individuals with the highest fitness score, indicating the best RF performance (e.g., area under the receiver operating characteristic [AUROC]), (4) crossover, and (5) mutation for new individuals in the next generation (Supplementary Figure S2).

In the first step of miRDM-rfGA, we generated the initial population to undergo successive evolution through the GA. A large number of individuals with diverse solutions within the initial population is necessary for successfully deriving optimal solutions. Therefore, we randomly generated 1,000 individuals, limited by our computational power available. The randomly generated 1000 individuals have diverse solutions for accessing the miRNA expression dataset. Within the initial population, each individual carried a specific number of miRNAs (described as “genes”) as features (Supplementary Figure S2A). The features of each individual were mapped to 1139 miRNA indices in the pre-processed dataset. In other words, an individual (*ind*) consists of 1139 features. the features were represented as *ind* = (x_1_, x_2_, …, x_1139_). x_i_ has a binary variable (i.e., {0,1}) where 1 indicates selection of the *i*-th miRNA and 0 indicates non-selection of the miRNA in the dataset. Subsequently, miRDM-rfGA randomly selects features from the 1139 indices according to the predefined number of selected features for each individual in the initial population (Supplementary Figure S2A). Then, given the individual, the expression profiles of the selected features (miRNAs) were extracted from the miRNA-Seq input data (i.e., the expression matrix of samples by miRNAs), generating a subset of the input data as a training set for the RF model. With the selected features in each individual, we calculated a fitness score as follows (fitness function) (Supplementary Figure S2B):

1 $$Fitness=100\times \frac{\sum_{k=1}^{M}{AUC}_{k}}{M}-W \times \left|x-b\right|$$

where *AUC*_*k*_ is the AUROC from the RF model for classifying T2DM and HC in the *k*-th fold during *M* fold cross-validation in the training set; $x$ is the number of selected features (miRNAs) in the individual; *W* is the penalty weight; and *b* is the optimal number of selected genes for the optimal biomarker combination.

Each individual was evaluated with a fitness score, and the individual with the highest fitness score was included in the population. The individual was then included in the next generation, and the other individuals in the next generation were produced by crossover and mutation (Supplementary Figure S2C). miRDM-rfGA iterates these phases for *G* generations and derives the best individual among these generations (Supplementary Figure S2D). Based on miRDM-rfGA, we created six parameter settings (settings 1 to 6), and the parameters *N*, *W*, *b*, *G*, crossover rate, and mutation rate are listed in Table 1. For the GA process, “DEAP” v.1.3.1 in Python 3.7 were used [40].

**Table 1:** Description of miRDM-rfGA parameter settings and the mean AUROC score in each setting

GA settings	Setting 1	Setting 2	Setting 3	Setting 4	Setting 5	Setting 6
The number of selected features in the initial individuals (*N*)	3	6	9	12	9	12
The optimal number of selected features (*b*)	3	3	3	3	3	3
Population	1000	1000	1000	1000	1000	1000
Generation (*G*)	200	200	200	200	500	300
Crossover rate	0.8	0.8	0.8	0.8	0.8	0.8
Mutation rate	0.003	0.003	0.003	0.003	0.003	0.003
Penalty weight (*W*)	20	15	10	7	10	7
Generation consisting of the best individual	168	31	58	139	463	44
Selected miRNAs	hsa-miR-29b-1-5p, hsa-miR-6738-3p, and hsa-miR-125b-2-3p	hsa-miR-494-3p, hsa-miR-668-3p, and hsa-miR-29b-1-5p	hsa-miR-222-5p, hsa-miR-671-5p, and hsa-miR-1307-3p	hsa-miR-494-3p, hsa-let-7b-5p, and hsa-miR-29b-1-5p	hsa-let-7b-5p, hsa-miR-125b-5p, and hsa-miR-7-5p	hsa-miR-7-5p, hsa-miR-92b-3p, and hsa-let-7b-5p
Fold for cross-validation of test data	3	3	3	3	3	3
Mean AUROC score by threefold cross-validation in test set and standard deviation	0.86 ± 0.09	0.89 ± 0.08	0.87 ± 0.05	0.89 ± 0.06	0.92 ± 0.04	0.90 ± 0.06

For traditional feature selection using the F-test in ANOVA, scikit-learn's f_classif function was used. Feature importance was calculated for each feature using f_classif, the top three miRNAs with the highest feature importance were selected through SelectKBest (k = 3), and the RF method was used to evaluate the discrimination power of T2DM for the selected miRNA biomarker set (setting 7).

For Lasso, we applied logistic regression using L1-regularization and used the SelectedFromModel (k = 3) function. Then, Lasso (setting 8) and RF (setting 9) were used as models for classifying T2DM using the selected miRNA biomarker set. Detailed information on settings 7, 8, and 9 is presented in Table 2.

**Table 2:** Description of the traditional feature selection methods and the mean AUROC score in each setting

Traditional feature selection methods	Setting 7	Setting 8	Setting 9
Feature selection models	Univariate feature selection (f_classif)	Lasso (Logistic regression using L1 regularization)	Lasso (Logistic regression using L1 regularization)
Selection methods	SelectKBest (top 3)	SelectFromModel (top 3)	SelectFromModel (top 3)
T2DM classification model	Random forest	Lasso	Random forest
Selected miRNAs	hsa-miR-6820–5p, hsa-miR-29b-2-5p, and hsa-miR-1307-3p	hsa-miR-22-3p, hsa-miR-92a-3p, and hsa-miR-181a-5p	hsa-miR-22-3p, hsa-miR-92a-3p, and hsa-miR-181a-5p
Fold for cross-validation of test data	3	3	3
Mean AUROC score by threefold cross-validation in test set and standard deviation	0.72 ± 0.08	0.64 ± 0.05	0.52 ± 0.02

### Performance comparison

For each trained model in each setting, the mean AUROC score was calculated using threefold cross-validation in the test set. The performance of each setting was compared using the mean AUROC.

### Principal component analysis

For the three best miRNA biomarker sets, we devised a method to distinguish between T2DM and HC using the most optimum miRNA biomarker set. Therefore, principal component analysis (PCA) was performed with three components using the miRNA-Seq data.

### Target mRNA identification and pathway enrichment analysis

MIENTURNET (http://userver.bio.uniroma1.it/apps/mienturnet/, accessed on 12 April, 2021) [41] was used for identifying the putative target genes of the differentially expressed circulating miRNAs and analyzing their pathway enrichment.

### Correlation analysis in mouse population data

The purpose of this analysis was to determine the correlation between diabetes-related clinical indicators and gene expression targeted by selected miRNAs using publicly available mouse population data [27]. For this analysis, two gene expression datasets in mouse liver tissue were obtained from the BxD mouse high-fat diet (HFD) [EPFL/LISP BXD HFD Liver Affy Mouse Gene 1.0 ST (Aug 18) RMA] and chow diet (CD) [EPFL/LISP BXD CD Liver Affy Mouse Gene 1.0 ST (Aug 18)] cohort from the GeneNetwork 1 database (http://gn1.genenetwork.org) [27].

The mRNA expression levels of *IGF1R, IRS2,* and *PIK3CD* were confirmed from mRNA gene expression array data in the liver tissue of the HFD and CD cohorts. In the gene expression data, we compared the top 25% of each gene with the bottom 25% of the group and clinical parameters.

Spearman’s correlation analysis was performed between gene expression data and the clinical parameters insulin response (IR) and oral glucose tolerance test (OGTT).

### Statistical analysis

Student’s *t*-test was used for analysis of differentially expressed miRNAs and DE genes in BxD mouse data between groups (T2DM vs. HC). For the DE analysis in the GEO gene and miRNA expression array dataset, we used GEO2R [42]. Correlations were evaluated using Spearman’s correlation coefficient. All reported *P* values were statistically significant when less than 0.05.

## Results

### Performance of feature selection using miRDM-rfGA and traditional feature selection

In this study, we identified the optimal miRNA biomarker combination. For this, we used mirDM-rfGA and traditional feature selection methods and evaluated the prediction performance of the miRNA subsets derived from each method. The workflow for determining the optimal miRNA features is presented in Fig. 1.

**Fig. 2:**
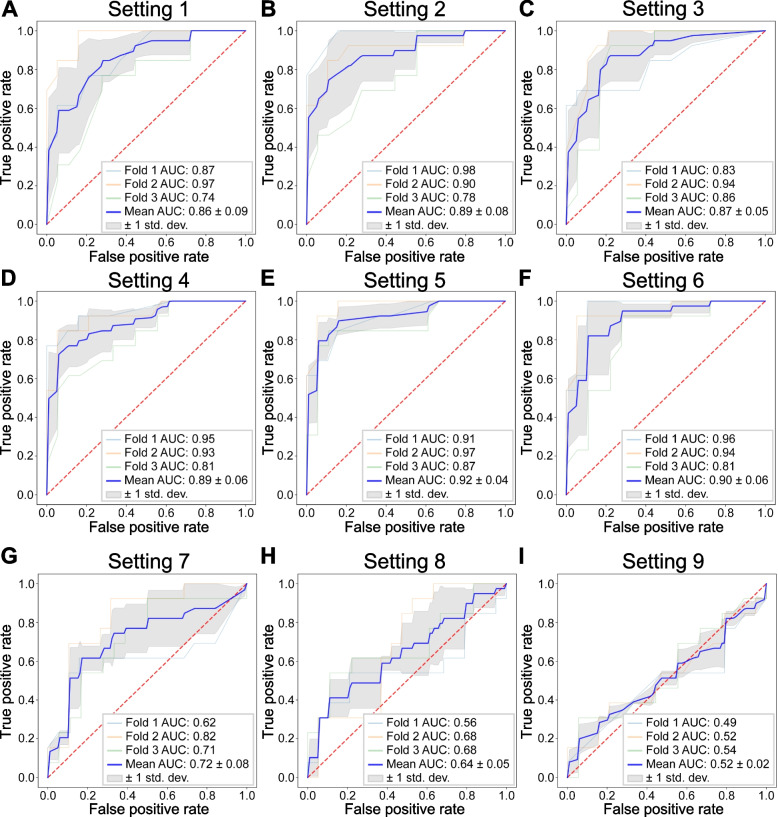
Performance of classifying T2DM and HC using selected miRNA biomarker set from nine feature selection settings. **A** Settings 1, (**B**) 2, (**C**) 3, (**D**) 4, (**E**) 5, and (**F**) 6 were configured based on miRDM-rfGA. **G** Settings 7, (**H**) 8, and (**I**) 9 were configured using traditional feature selection methods (F-test and Lasso). In the test set, threefold cross validation was used, and mean AUROC and standard deviation was calculated

First, we derived the three miRNA subsets using mirDM-rfGA under six settings (settings 1 to 6) (Fig. 2A, B, C, D, E, F and Table 1). Using each miRNA subset data derived from each setting, we constructed an RF model classifying T2DM patients. Each RF model was compared with the mean AUROC (± 1 standard deviation) through threefold cross-validation using the test set.

Among the six settings using mirDM-rfGA, setting 5 showed the best performance. The AUROC of the model was calculated through the test set, and the mean AUROC was 0.92 ± 0.04. The miRNAs subset using setting 5 included 'hsa-let-7b-5p,' 'hsa-miR-125b-5p,' and 'hsa-miR-7-5p,' (Fig. 2E and Table 1).

We also applied traditional feature selection using univariate feature selection methods (F-test in ANOVA and Lasso; settings 7 to 9; Fig. 2G, H, I and Table 2) for comparison with the settings using mirDM-rfGA.

As a result, the mean AUROC value of setting 7 was 0.72 ± 0.08 ('hsa-miR-6820-5p,' 'hsa-miR-29b-2-5p,' and 'hsa-miR-1307-3p'); that of setting 8 was 0.64 ± 0.05 ('hsa-miR-22-3p,' 'hsa-miR-92a-3p,' and 'hsa-miR-181a-5p'), and that of setting 9 was 0.52 ± 0.02 ('hsa-miR-22-3p,' 'hsa-miR-92a-3p,' and 'hsa-miR-181a-5p') (Table 2).

In summary, the setting 5 using mirDM-rfGA showed the best performance in detecting T2DM, and the miRNA set derived by the setting 5 included 'hsa-let-7b-5p,' 'hsa-miR-125b-5p,' and 'hsa-miR-7-5p'.

**Fig. 3:**
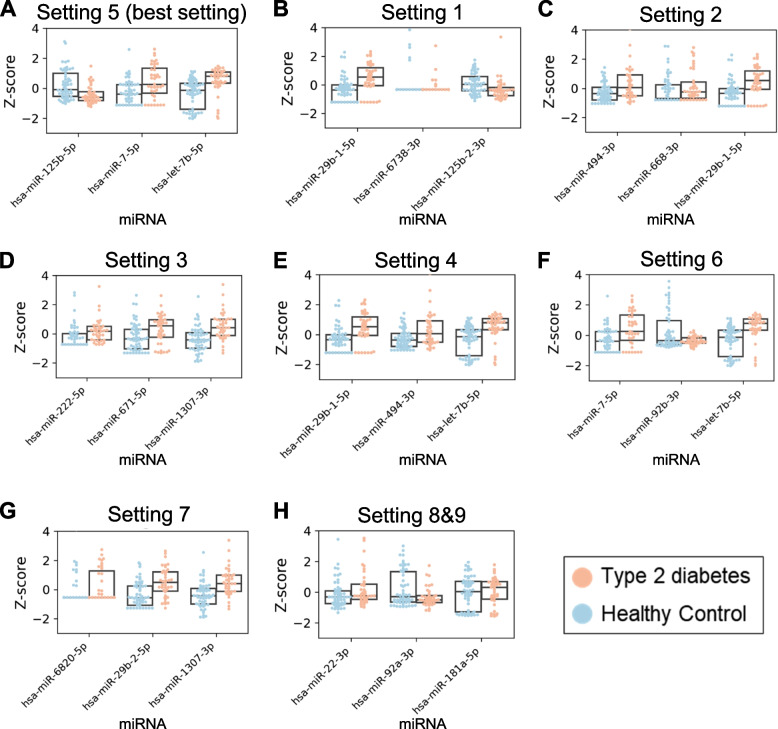
Difference of miRNA biomarker expression levels between T2DM and HC group. We derived miRNA biomarker set using each setting for feature selection. We compared miRNA expression levels between T2DM and HC groups. **A** Settings 5 (the best setting), (**B**) 1, (**C**) 2, (**D**) 3, (**E**) 4, and (**F**) 6 were configured based on miRDM-rfGA. **G** Setting 7, and (**H**) settings 8 and 9 were configured using traditional feature selection methods (F-test and Lasso). The same miRNAs were selected in settings 8 and 9. Each miRNA expression was converted into z-score

The log2 fold change of T2DM vs. HC for each miRNA in setting 5 was 0.468, − 0.853, and 0.953, respectively (Fig. 3A and Table 3). The *P* values were also statistically significant at 4.33e − 5, 4.59e − 4, and 1.75e − 4, respectively. The DE analysis results and statistical significance of miRNAs derived from other settings are described in Fig. 3B–H and Table 3.

**Table 3:** DE analysis of miRNA biomarkers derived by each setting

Feature selection settings	Selected miRNA	^a^LogFC (T2DM vs. HC)	*P* value
Setting 1	hsa-miR-29b-1-5p	0.947	< 0.001
	hsa-miR-494-3p	0.912	0.001
	hsa-let-7b-5p	0.468	< 0.001
Setting 2	hsa-miR-29b-1-5p	0.947	< 0.001
	hsa-miR-6738-3p	-1.400	0.201
	hsa-miR-125b-2-3p	-0.768	0.007
Setting 3	hsa-miR-222-5p	1.083	0.006
	hsa-miR-671-5p	0.593	0.007
	hsa-miR-1307-3p	0.535	< 0.001
Setting 4	hsa-miR-494-3p	0.912	0.001
	hsa-miR-668-3p	0.506	0.182
	hsa-miR-29b-1-5p	0.947	< 0.001
Setting 5	hsa-let-7b-5p	0.468	< 0.001
	hsa-miR-125b-5p	-0.853	< 0.001
	hsa-miR-7-5p	0.953	< 0.001
Setting 6	hsa-miR-7-5p	0.953	< 0.001
	hsa-miR-92b-3p	-0.837	0.002
	hsa-let-7b-5p	0.468	< 0.001
Setting 7	hsa-miR-6820-5p	1.132	0.041
	hsa-miR-29b-2-5p	0.961	< 0.001
	hsa-miR-1307-3p	0.535	< 0.001
Setting 8 & 9	hsa-miR-22-3p	0.244	0.068
	hsa-miR-92a-3p	-0.520	0.008
	hsa-miR-181a-5p	0.085	0.645

^a^*LogFC* Log_2_FoldChange, *T2DM* Type 2 diabetes, *HC* Healthy control

### The relationship between the best miRNA biomarker set and their target mRNAs

**Fig. 4:**
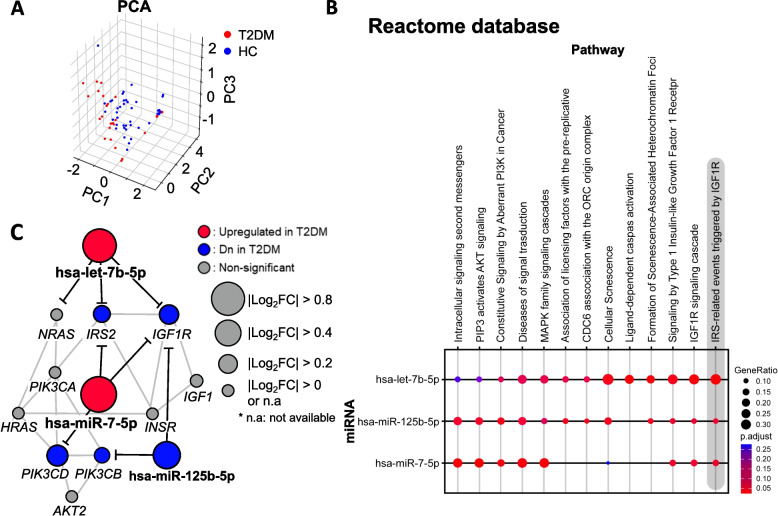
The best miRNA biomarker set in setting 5. **A** Principal Component Analysis (**B**) Functional enrichment analysis of the miRNA biomarker set. **C** Pathway of IRS-related events triggered by IGF1R signaling. DE analysis result of the best miRNA biomarker set and the targeted mRNAs in skeletal muscle. The circle size of each point is proportional to the |log2 of fold change in T2DM vs. healthy control|

We used PCA to determine how the optimal miRNA biomarker subset (hsa-miR-125b-5p, hsa-miR-7-5p, and hsa-let-7b-5p) distinguished between T2DM patients and HCs. The T2DM and HC groups were differentiated using only the expression levels of the three miRNAs (Fig. 4A). After confirming that these three miRNAs can discriminate against the diabetic group, functional enrichment analysis of the miRNA-target gene was conducted based on the miRTarbase database [43, 44] to determine the signaling pathway to which the target genes of the three miRNAs belong. All three miRNAs were found to be most enriched in insulin receptor substrate (IRS)-related events triggered by IGF1R in the Reactome database (Fig. 4B) [45].

In this pathway, the target genes were *PIK3CB*, *IGF1R* targeted by hsa-miR-125b-5p, *IRS2*, *IGF1R*, and *PIK3CD* targeted by hsa-miR-7-5p, and *IRS2* and *IGF1R* targeted by hsa-let-7b-5p.

Interestingly, all three miRNAs target *IGF1R* in the pathway network (Fig. 4B).

Next, we confirmed the expression patterns of the genes targeted by the miRNA biomarker in a public dataset of T2DM studies.

In the skeletal muscle tissue (GSE22309), we also confirmed that the gene expression levels of *IRS2*, *IGF1R*, and *PIK3CD* were decreased in patients with T2DM after insulin treatment compared with those in healthy participants after insulin treatment (Fig. 4C and Supplementary Table S2). However, the regulation pattern of hsa-miR-125b-5p on its targets (*IGF1R* and *PIK3CB*) was not observed (Fig. 4C).

### Correlation with diabetes-related clinical variables according to the gene expression targeted by the miRNA biomarkers

We also investigated the correlation between mRNA targeted by miRNA biomarkers in IRS-related events by the IGF1R pathway and clinical indicators related to diabetes in a publicly available BxD mouse database [27].

**Fig. 5:**
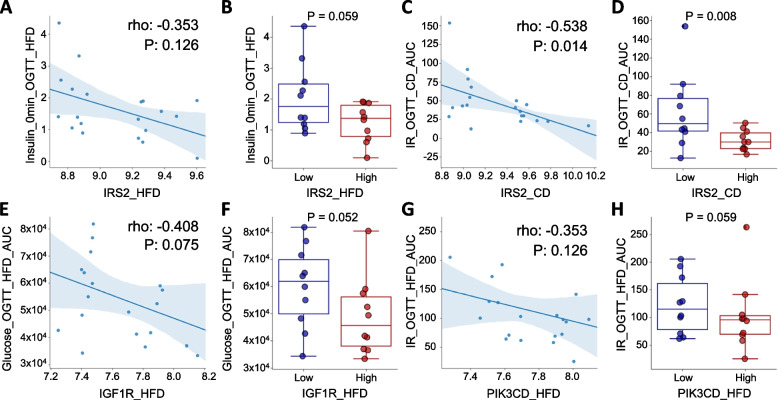
Correlation and DE analysis between targeted mRNAs and clinical parameters related to T2DM, using public BxD mouse database: GeneNetwork. **A** Spearman’s correlation analysis between hepatic IRS2 expression (x-axis) in BxD high-fat diet (HFD) mice and Insulin (y-axis) during OGTT and (**B**) the comparison between the highest 25% mice (HFD) of *IRS2* gene expression with the bottom 25% mice (HFD) of the gene expression. **C** Spearman’s correlation analysis between hepatic *IRS* expression in BxD chow diet (CD) mice and insulin response (IR) during OGTT and (**D**) the comparison between the top 25% mice (CD) of *IRS2* gene expression with the bottom 25% mice (CD) of the gene expression. **E** Spearman’s correlation analysis between hepatic *IGF1R* expression level in BxD HFD mice and glycemia level during OGTT and (**F**) the comparison between the top 25% mice (HFD) of *IGF1R* gene expression with the bottom 25% mice of the gene. **G** Spearman’s correlation analysis between hepatic *PIK3CD* expression level in BxD HFD mice and IR during OGTT and (**H**) the comparison between the top 25% mice (HFD) of *PIK3CD* gene expression with the bottom 25% mice (HFD) of the gene

Correlation analysis was performed using the GeneNetwork BxD mouse database. Among various clinical variables, when the expression levels of *IGF1R*, *PIK3CD*, and *IRS2* were low in HFD and CD mice, both glucose levels in the OGTT and IR values during OGTT increased, and these results showed a negative correlation (Fig. 5 and Supplementary Table S3).

## Discussion

In this study, we identified a miRNA biomarker set consisting of three miRNAs for detecting T2DM using public miRNA-Seq data. To this end, we devised miRDM-rfGA, which is an GA-based feature selection algorithm, and created biomarker discovery settings using GA (six settings) and traditional feature selection methods (3 settings using F-test and Lasso) by constructing RF classifier models to detect T2DM, using miRNAs obtained through each setting. We then compared the classification performance of the biomarker sets from all settings. As a result, setting 5 using miRDM-rfGA (mean AUROC: 0.92) outperformed traditional feature selection approaches in identifying these biomarkers. From the setting 5, we determined that a set of biomarkers consisting of three miRNAs (hsa-miR-125b-5p, hsa-miR-7-5p, and hsa-let-7b-5p) had the most optimal classification performance for T2DM (Fig. 2E). To confirm the association between the discovered miRNA biomarker set and diabetes, we performed a functional enrichment analysis of miRNA-targeted mRNAs. As a result, IRS-related signaling by IGF1R, which is directly related to diabetes, was derived (*IRS2*, *IGF1R*, PIK3CD, and *PIK3CB*) (Fig. 4C). In addition, correlation analysis was performed between the expression levels of the corresponding mRNA and clinical variables related to the detection of diabetes using the public data of BxD mice (Fig. 5) [27]. These results confirmed that the gene expression levels in diabetes were consistent with those from our study.

In addition, we confirmed that there are various studies supporting that three miRNAs (hsa-miR-125b-5p, hsa-miR-7-5p, and hsa-let-7b-5p) are related to diabetes.

Let-7b-5p is a miRNA belonging to the let-7 family, which has a common seed region up to nucleotides 2–8, and is known to perform similar functions [46]. In this study, the relationship between let-7 and diabetes showed that glucose tolerance was inhibited in let-7 overexpressing mice, and let-7 knockdown via let-7 anti-miR restored glucose intolerance caused by obesity [46]. In addition, functional recovery of insulin signaling was observed in the muscle and liver following let-7 anti-miR treatment [46]. In another study, a Lin28 transgenic mouse model showed that let-7 downregulation effectively reduced glucose levels in a high-fat diet and activated the Insulin-PI3K-mTOR signaling pathway through let-7 suppression [47]. In our study (Fig. 3E), let-7b-5p was overexpressed in the group of patients with T2DM as compared to that in the HCs. The results of this study confirmed that the overexpression of let-7 was consistent with the study results of miRNAs adversely affecting glucose tolerance and insulin sensitivity.

miR-7 is known as a miRNA related to insulin secretion and glucose homeostasis in the insulin signaling pathway. Agbu et al. reported that circulating glucose and triglyceride levels were increased in Drosophila insulin-producing cells (IPCs) overexpressing miR-7 as compared to those in wild-type cells [48]. Additionally, miR-7-5p inhibits glucose uptake and insulin-induced AKT phosphorylation by affecting the downregulation of IRS-1 and IRS-2 [16, 49]. Expression patterns of miR-7-5p and IRS2 were also observed in the present study (Figs. 3E and 4C).

According to Gong et al., the expression of hsa-miR-125b-5p is downregulated in the retinas of rats with STZ-induced diabetes [50]. They also confirmed that miR-125b-5p was downregulated with the progression of diabetic retinopathy (DR), suggesting that hsa-miR-125b-5p might be used as an effective T2DM and DR treatment, and the expression pattern of hsa-miR-125b-5p was identical to that observed in our study (Fig. 3E).

The genes targeted by the three miRNAs for the optimal miRNA biomarker set of T2DM were *IRS2*, *IGF1R*, and *PIK3CD*. *IRS2* is an insulin receptor substrate 2 involved in insulin sensitivity as an insulin signaling pathway, and whose expression levels are decreased in T2DM [51]. In a mouse model study, when IRS2 was knocked out, obesity and insulin sensitivity decreased. As a result, glucose tolerance was induced and developed into T2DM. This led to hyperinsulinemia and β-cell damage [52, 53].

IGF1R functions as an insulin receptor by forming a dimer with the insulin receptor as an insulin-like growth factor-1 receptor [54]. Dong et al. confirmed that the expression level of IGF1R was decreased in the liver of diabetic rats, and the regulation of this gene by miRNAs could play a role in the improvement of insulin resistance [55]. Razny et al. compared the expression levels of whole blood mRNA between an obese group with insulin resistance and an obese group without insulin resistance [56], and found that the gene expression level of *IGF1R* had decreased. Therefore, the decreasing pattern in the gene expression level of *IGF1R* coincided with the increasing pattern in the expression level of hsa-let-7b-5p and hsa-miR-7-5p targeting IGF1R.

PIK3CD refers to Phosphatidylinositol-4,5-Bisphosphate 3-Kinase Catalytic Subunit delta, which is a catalytic subunit of PI3K. PIK3CD participates in PI3-Kinase signaling and affects the AKT pathway, and the gene expression level of *PIK3CD* is diminished in the skeletal muscle tissue of diabetic patients [57]. In addition, inhibition of PI3K signaling in skeletal muscle tissue in mouse models results in insulin resistance and systemic glucose intolerance. Further, free fatty acid and triglyceride levels in the blood are elevated [58]. Insulin resistance occurs when PI3K signaling is inhibited [59, 60] and has been observed in adipocytes [61, 62], muscle cells [57], the liver [63], and blood [56]. Du et al. reported that PIK3CD-targeting miRNA can inhibit the insulin signaling pathway and thus become a target gene that can regulate insulin resistance [63].

By devising miRDM-rfGA, we identified a set of putative diagnostic and treatment biomarkers for T2DM using GA. Our study was limited by the number of samples (95). However, to compensate for this limitation, we used correlation analysis with the public BxD mouse database to confirm that the mRNAs targeted by the miRNA biomarker set were also related to T2DM [27]. In addition, our study was limited to discovering diagnostic and treatment biomarkers composed of three miRNAs. Based on this, an extended follow-up study may be conducted.

In addition, while our method could have allowed for an extended study into diabetic complications along with diabetes, our study was also focused on only type 2 diabetes. However, using a publicly available miRNA dataset (GEO accession: GSE51674) covering ‘diabetic nephropathy with type 2 diabetes’ (T2DN) in kidney tissue (Supplementary Table S1), hsa-let-7b, hsa-miR-125b were up-regulated in T2DN (Supplementary Table S4 and Supplementary Figure S3). Among these miRNAs, we may indicate hsa-let-7b as a biomarker candidate not only for T2DM but also for T2DN.

This study suggested that the feature selection method combining genetic algorithms (GA) and machine learning is superior to traditional feature selection methods, and also that this approach can be useful in deriving optimal biomarker combinations that can be applied across various diseases.

## Conclusions

We derived an optimal miRNA biomarker set that could detect T2DM using GA to process miRNA-Seq data. Thus, GA can be used as an effective method for biomarker discovery.

### Supplementary Information

**Additional file 1: Table S1.** Description of public miRNA-Seq and gene expression array data used in this study.** Table S2.** GEO2R analysis of mRNAs (PIK3CD, IGF1R, IRS2, NRAS, and PIK3CB) targeted by the best miRNA biomarker set in skeletal muscle tissue (GSE22309). **Table S3.** Spearman correlation matrix between hepatic mRNA expression of the targeted genes in BxD mouse and clinical parameters related to type 2 diabetes.** Table S4.** Differential expression analysis of miRNAs (hsa-let-7b, hsa-miR-125b, and hsa-miR-7) in kidney tissues of T2DN vs. HC (GEO accession: GSE51674).** Fig. S1.** Prisma diagram of miRNA-Seq dataset acqusition in this study. In SRA, we acquired 2 datasets of type 2 diabetes study in blood tissue. Hence, 95 samples were used for biomarker discovery.** Fig. S2.** Workflow of miRDM-rfGA for biomarker discovery of optimal miRNA to classify T2DM and HC. (A) In the initial population, We generated 1000 individuals, and features in each individual are mapped to 1139 miRNA indices. miRDM-rfGA randomly select features among 1139 miRNA. (B) Fitness score is calculated for each individual based on AUROC score from RF classifier, then (C) miRDM-rfGA chooses the individual with the highest fitness score. The individual is included in the next generation and the other individuals are produced for next generation. Crossover and mutation are applied during the production of new individuals. (D) miRDM-rfGA iterates these phases for the number of generation G and derives the best individual among G generations.** Fig. S3.** Difference of the selected miRNA biomarkers expression levels between ‘diabetic nephropathy with type 2 diabetes’ (T2DN) and HC group. We obtained a publicly available miRNA expression dataset (GEO accession: GSE51674) to inspect the possibility of prediction of the risk of diabetic complications. We compared the selected miRNA expression levels between T2DN and HC groups.

## Data Availability

The datasets presented in this study can be found in online repositories. The names of the repository/repositories and accession number(s) can be found below: NCBI Sequence Read Archive (SRA), accession no: PRJNA476995 (https://www.ncbi.nlm.nih.gov/bioproject/PRJNA476995) and PRJNA354381 (https://www.ncbi.nlm.nih.gov/bioproject/PRJNA354381); NCBI Gene Expression Omnibus (GEO), accession no: GSE22309 (https://www.ncbi.nlm.nih.gov/geo/query/acc.cgi?acc=GSE22309) and GSE51674 (https://www.ncbi.nlm.nih.gov/geo/query/acc.cgi?acc=GSE51674). The source code for the miRDM-rfGA in setting 5 is available at GitHub (https://github.com/labnams/miRDM-rfGA).

## Abbreviations

*T2DM*: Type 2 diabetes mellitus
*HC*: Healthy control
*miRNA*: MicroRNA
*SRA*: NCBI Sequence Read Archive
*GEO*: NCBI Gene Expression Omnibus
*R**PM*: Reads per million
*DE*: Differentially expressed
*GA*: Genetic algorithm
*AUROC*: Area under receiver operating characteristic
*P**CA*: Principal component analysis
*R**F*: Random forest
*HFD*: High fat diet
*CD*: Chow diet
*IR*: Insulin response
*OGTT*: Oral glucose tolerance test
*IPC*: Drosophila insulin production cell
*DR*: Diabetic retinopathy
*T2DN*: Diabetic nephropathy with type 2 diabetes
