# Supplementary material for: miRDM-rfGA: Genetic algorithm-based identification of a miRNA set for detecting type 2 diabetes
Source: BMC Med Genomics. 2023 Aug 22;16:195. doi: 10.1186/s12920-023-01636-2 (PMC10463588; doi:10.1186/s12920-023-01636-2)
Supplement: Supplementary file 1 — Additional file 1: Table S1. Description of public miRNA-Seq and gene expression array data used in this study. Table S2. GEO2R analysis of mRNAs (PIK3CD, IGF1R, IRS2, NRAS, and PIK3CB) targeted by the best miRNA biomarker set in skeletal muscle tissue (GSE22309). Table S3. Spearman correlation matrix between hepatic mRNA expression of the targeted genes in BxD mouse and clinical parameters related to type 2 diabetes. Table S4. Differential expression analysis of miRNAs (hsa-let-7b, hsa-miR-125b, and hsa-miR-7) in kidney tissues of T2DN vs. HC (GEO accession: GSE51674). Fig. S1. Prisma diagram of miRNA-Seq dataset acqusition in this study. In SRA, we acquired 2 datasets of type 2 diabetes study in blood tissue. Hence, 95 samples were used for biomarker discovery. Fig. S2. Workflow of miRDM-rfGA for biomarker discovery of optimal miRNA to classify T2DM and HC. (A) In the initial population, We generated 1000 individuals, and features in each individual are mapped to 1139 miRNA indices. miRDM-rfGA randomly select features among 1139 miRNA. (B) Fitness score is calculated for each individual based on AUROC score from RF classifier, then (C) miRDM-rfGA chooses the individual with the highest fitness score. The individual is included in the next generation and the other individuals are produced for next generation. Crossover and mutation are applied during the production of new individuals. (D) miRDM-rfGA iterates these phases for the number of generation G and derives the best individual among G generations. Fig. S3. Difference of the selected miRNA biomarkers expression levels between ‘diabetic nephropathy with type 2 diabetes’ (T2DN) and HC group. We obtained a publicly available miRNA expression dataset (GEO accession: GSE51674) to inspect the possibility of prediction of the risk of diabetic complications. We compared the selected miRNA expression levels between T2DN and HC groups. [file 12920_2023_1636_MOESM1_ESM.docx]

**miRDM-rfGA: Genetic Algorithm-Based Identification of a MiRNA Set for Detecting Type 2 Diabetes**

Park *et al*.

**Supplementary Information**

Supplementary Tables S1 through S4

Supplementary Figures S1 and S3

Supplementary References

**Supplementary Table S1.** Description of public miRNA-Seq and gene expression array data used in this study.

| **SRA project ID**  **(miRNA-Seq)** | **Source** | **No. of control subjects** | **No. of case patients** | **References** |
| --- | --- | --- | --- | --- |
| PRJNA476995 | Blood | 40 | 32 (T2DM) |  |
| PRJNA354381 | Blood | 16 | 7 (T2DM) | - [1] |
| **GEO dataset ID**  **(mRNA or miRNA expression)** | **Source** | **No. of control subjects** | **No. of case patients** | - **References** |
| GSE22309 (mRNA) | Skeletal muscle | 20 (Insulin treated control) | 15 (Insulin treated diabetes) | [2, 3] |
| GSE51674 (miRNA) | Kidney | 4 (HC) | 6 (T2DN) | [4] |

T2DM: Type 2 Diabetes Mellitus; T2DN: Diabetic nephropathy with type 2 diabetes

**Supplementary Table S2.** GEO2R analysis of mRNAs (*PIK3CD*, *IGF1R*, *IRS2, NRAS, and PIK3CB*) targeted by the best miRNA biomarker set in skeletal muscle tissue (GSE22309).

| **ID** | **Gene Symbol** | **Gene name** | **logFC**  ***(T2DM vs. HC)** | **P Value** |
| --- | --- | --- | --- | --- |
| 32121_at | *PIK3CD* | phosphatidylinositol-4,5-bisphosphate 3-kinase catalytic subunit delta | -0.241 | <0.001 |
| 31335_at | *IGF1R* | insulin like growth factor 1 receptor | -0.201 | 0.008 |
| 40180_at | *IRS2* | insulin receptor substrate 2 | -0.164 | 0.025 |
| 31699_at | *PIK3CB* | phosphatidylinositol-4,5-bisphosphate 3-kinase catalytic subunit beta | -0.140 | 0.025 |
| 1539_at | *NRAS* | neuroblastoma RAS viral oncogene homolog | 0.027 | 0.747 |

***** T2DM: Type 2 Diabetes Mellitus, HC: Healthy Control.

**Supplementary Table S3.** Spearman correlation matrix between hepatic mRNA expression of the targeted genes in BxD mouse and clinical parameters related to type 2 diabetes.

| **mRNA expression data** | **Phenotype data of BxD mouse** | **Spearman_Rho** | **Spearman_Pval** |
| --- | --- | --- | --- |
| Igf1r_EPFL/LISP BXD HFD Liver Affy Mouse Gene 1.0 ST (Aug18) RMA | Metabolism: Glycemia during oral glucose tolerance test (OGTT), overall, high fat diet (60% kCal/fat HarlanTD.06414), males [AUC] (EPFL LISP3 Cohort) | -0.408 | 0.075 |
| Pik3cd_EPFL/LISP BXD HFD Liver Affy Mouse Gene 1.0 ST (Aug18) RMA | Metabolism: Insulin response from oral glucose tolerance test (OGTT), at 17 weeks of age, high fat diet (60% kCal/fat HarlanTD.06414), males [AUC] (EPFL LISP3 Cohort) | -0.335 | 0.148 |
| Irs2_EPFL/LISP BXD HFD Liver Affy Mouse Gene 1.0 ST (Aug18) RMA | Metabolism: Insulin during oral glucose tolerance test (OGTT), 0min, at 17 weeks of age, (after overnight fast), high fat diet (60% kCal/fat HarlanTD.06414), males [ug/mL] (EPFL LISP3 Cohort) | -0.353 | 0.126 |
| Irs2_EPFL/LISP BXD CD Liver Affy Mouse Gene 1.0 ST (Aug18) RMA | Metabolism: Insulin response from oral glucose tolerance test (OGTT), at 17 weeks of age, chow diet (6% kCal/fat Harlan.2918), males [AUC] (EPFL LISP3 Cohort) | -0.538 | 0.014 |

**Supplementary Table S4.** Differential expression analysis of miRNAs (hsa-let-7b, hsa-miR-125b, and hsa-miR-7) in kidney tissues of T2DN vs. HC (GEO accession: GSE51674).

| **miRNA** | **logFC**  **(T2DN vs. HC)** | **P Value** |
| --- | --- | --- |
| hsa-let-7b | 1.353 | 0.001 |
| hsa-miR-125b | 1.292 | 0.003 |
| hsa-miR-7 | -0.518 | 0.500 |

T2DN: Diabetic nephropathy with type 2 diabetes; HC: Healthy Control.

**Supplementary Figure S1.** Prisma diagram of miRNA-Seq dataset acqusition in this study. In SRA, we acquired 2 datasets of type 2 diabetes study in blood tissue. Hence, 95 samples were used for biomarker discovery.


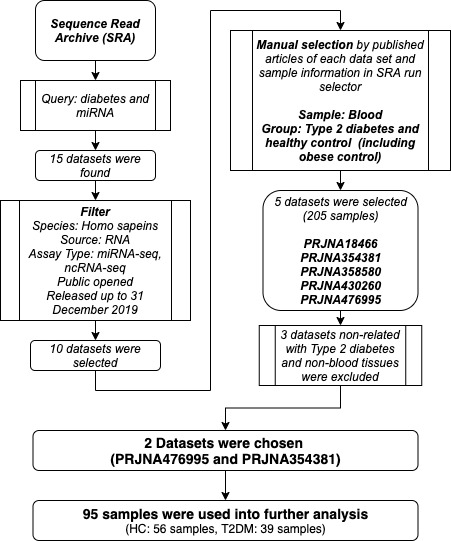


**Supplementary Figure S2**. Workflow of miRDM-rfGA for biomarker discovery of optimal miRNA to classify T2DM and HC. **(A)** In the initial population, We generated 1000 individuals, and features in each individual are mapped to 1139 miRNA indices. miRDM-rfGA randomly select features among 1139 miRNA. **(B)** Fitness score is calculated for each individual based on AUROC score from RF classifier, then **(C)** miRDM-rfGA chooses the individual with the highest fitness score. The individual is included in the next generation and the other individuals are produced for next generation. Crossover and mutation are applied during the production of new individuals. **(D)** miRDM-rfGA iterates these phases for the number of generation G and derives the best individual among G generations.


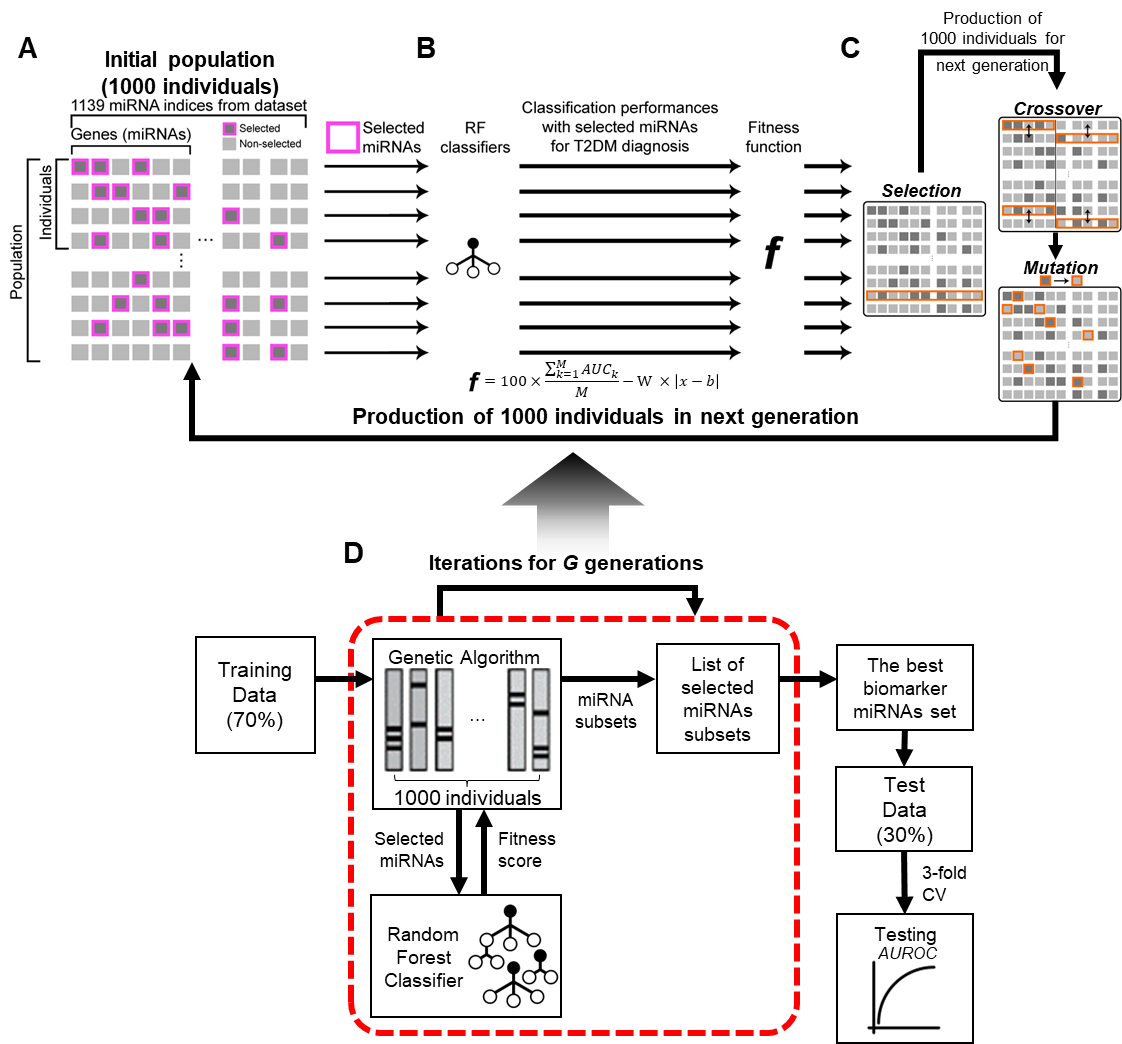


**Supplementary Figure S3. Difference of the selected miRNA biomarkers expression levels between ‘diabetic nephropathy with type 2 diabetes’ (T2DN) and HC group.** We obtained a publicly available miRNA expression dataset (GEO accession: GSE51674) to inspect the possibility of prediction of the risk of diabetic complications. We compared the selected miRNA expression levels between T2DN and HC groups.


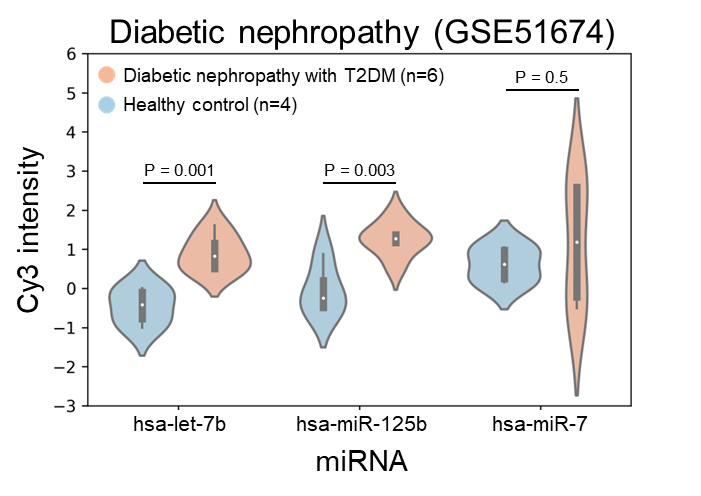


**Supplementary References**

1. Krauskopf J, de Kok TM, Schomaker SJ, Gosink M, Burt DA, Chandler P, et al. Serum microRNA signatures as "liquid biopsies" for interrogating hepatotoxic mechanisms and liver pathogenesis in human. PLoS One. 2017;12(5):e0177928-e.

2. Wu X, Wang J, Cui X, Maianu L, Rhees B, Rosinski J, et al. The effect of insulin on expression of genes and biochemical pathways in human skeletal muscle. Endocrine. 2007;31(1):5-17.

3. Wu X, Patki A, Lara-Castro C, Cui X, Zhang K, Walton RG, et al. Genes and biochemical pathways in human skeletal muscle affecting resting energy expenditure and fuel partitioning. J Appl Physiol (1985). 2011;110(3):746-55.

4. Conserva F, Barozzino M, Pesce F, Divella C, Oranger A, Papale M, et al. Urinary miRNA-27b-3p and miRNA-1228-3p correlate with the progression of kidney fibrosis in diabetic nephropathy. Scientific reports. 2019;9(1):11357.
